# Impact of CeO_2_-Doped Bioactive Glass on the Properties of CMC/PEG Hydrogels Intended for Wound Treatment

**DOI:** 10.3390/gels11121010

**Published:** 2025-12-16

**Authors:** Sofia Pacheco, Inês Alexandra Marques, Ana Salomé Pires, Maria Filomena Botelho, Sílvia Soreto Teixeira, Manuel Graça, Sílvia Gavinho

**Affiliations:** 1 i3N and Physics Department, University of Aveiro, 3810-193 Aveiro, Portugal; sofiapacheco@ua.pt (S.P.); silvia.soreto@ua.pt (S.S.T.); mpfg@ua.pt (M.G.); 2 Coimbra Institute for Clinical and Biomedical Research (iCBR) Area of Environment, Genetics and Oncobiology (CIMAGO), Institute of Biophysics, Faculty of Medicine, University of Coimbra, Azinhaga de Santa Comba, 3000-548 Coimbra, Portugal; pireslourenco@uc.pt (A.S.P.); mfbotelho@fmed.uc.pt (M.F.B.); 3 Center for Innovative Biomedicine and Biotechnology (CIBB), University of Coimbra, Rua Larga, 3004-504 Coimbra, Portugal; 4 Clinical Academic Center of Coimbra (CACC), Praceta Professor Mota Pinto, 3004-561 Coimbra, Portugal

**Keywords:** hydrogel, Bioglass 45S5, tissue regeneration, drug delivery, cerium, antioxidant

## Abstract

Diabetes mellitus is a serious public health problem, mainly due to the difficulty in healing chronic wounds, which present an inflammatory response for long periods of time and are more vulnerable to infections. Hydrogels are a promising therapeutic solution due to their biocompatibility, biodegradability, and ability to allow controlled release of therapeutic agents. The addition of bioactive glasses doped with therapeutic ions to hydrogels can also provide specific biological responses to the system and thus improve tissue regeneration. In this study, a hydrogel based on carboxymethylcellulose and polyethylene glycol with different degrees of crosslinking and enriched with 10% by weight of CeO_2_-doped Bioglass 45S5 was developed. Structural, morphological, mechanical, and biological characterizations were performed on bioactive glass, hydrogels, and hydrogels enriched with bioactive glass. Structural analyses confirmed the preservation of the typical amorphous structure of Bioglass 45S5, even after the incorporation of 5% molar CeO_2_, as well as the effectiveness of the polymer matrix crosslinking process. Structural analyses demonstrated the preservation of the typical amorphous structure of Bioglass 45S5, even after the incorporation of 5 mol% CeO_2_, as well as the effectiveness of the polymer matrix cross-linking process. The hydrogels exhibited distinct behaviours in terms of water absorption and degradation, showing that the sample with the lowest concentration of crosslinkers and bioactive glass allowed for a higher expansion rate and a higher degradation rate. The hydrogel with 10 wt% BG did not compromise cell viability and showed structural integrity after being subjected to cyclic flexible deformations, indicating its safety and suitability for use in tissue engineering.

## 1. Introduction

The skin is the largest organ in the human body and plays a vital role in various metabolic functions. Therefore, severe skin lesions can compromise health, leading to serious consequences in quality of life. Chronic wounds represent a significant challenge to health and the economy worldwide, especially in the context of diabetes mellitus. Chronic diabetic wounds are a consequence of unsuccessful healing, characterised by a persistent inflamed microenvironment characterised by excessive pro-inflammatory cytokines and high oxidative stress, an increased risk of infection and poor vascularisation [1].

According to the World Health Organization, diabetes currently affects around 830 million people worldwide [2]. The International Diabetes Federation (IDF) reports that 11.1% of adults aged 20 to 79 live with diabetes, accounting for approximately 3.4 million deaths in 2024. An estimated 43% of adults with diabetes remain undiagnosed, with nearly 90% residing in low- and middle-income countries. Forecasts indicate that the number of individuals affected will increase to 853 million by 2050. This global growing trend is closely related to the increase in the prevalence of chronic wounds, particularly diabetic foot ulcers. Due to reduced healing capacity, vascular dysfunction, and persistent inflammation associated with diabetes, chronic wounds have become one of its most serious and costly complications, emphasising the need for advanced and effective wound treatment strategies [3].

Conventional treatments for chronic wounds, such as drug administration, have unacceptable and ineffective results, which are associated with recurrent infections, high costs, and prolonged hospitalisation [4]. In addition, other approaches, such as hydrogel dressings, have been applied to protect and moisturise the wound site while absorbing exudates. These systems are typically based on natural or synthetic polymer networks, such as alginate, chitosan, gelatin, polyvinyl alcohol, or polyethylene glycol, which can retain large amounts of water and provide a moist environment conducive to tissue repair. However, commercially available hydrogels do not have any therapeutic action for the treatment of inflammatory processes or bacterial infections, which are highly prevalent in chronic diabetic wounds. Therefore, there is an urgent need to develop new therapeutic approaches to promote tissue regeneration and accelerate wound healing [5].

Recent studies have been dedicated to the development of bioactive hydrogels, introducing properties such as antibacterial, antioxidant, or angiogenic effects. Many hydrogels applied for therapeutic action can be formulated to respond to stimuli such as temperature, pH, enzymes, or reactive oxygen species [5]. This activation by external stimuli can alter their mechanical properties, expansion capacity, permeability and hydrophobicity, functioning as a controlled system for the drug administration or therapeutic agents, such as bioactive glass, which can actively promote tissue regeneration and accelerate wound healing [6].

Bioactive glasses (BG), particularly Bioglass 45S5, have attracted special attention because of their ability to release therapeutic ions that enhance cell metabolism, increase cell activity, and promote angiogenesis. Moreover, the ability to incorporate therapeutic ions, such as cerium, adds specific biological properties, promoting antioxidants and anti-inflammatory activity. When combined with hydrogels, the bioactive glass can improve the mechanical properties while also providing antibacterial activity at the wound site. This antibacterial activity is due to the release of ions, such as Ca^2+^ and Na^+^, which increase the local pH, thereby creating an alkaline environment hostile to bacterial growth [7,8,9]. Cerium has demonstrated considerable potential in the treatment of chronic diabetic wounds due to its unique redox behaviour, switching between the oxidation states Ce^3+^ and Ce^4+^, capable of effectively eliminating excess reactive oxygen species (ROS). The balance in the concentration of reactive oxygen species by cerium creates a microenvironment more favourable to fibroblast proliferation, collagen deposition and angiogenesis, all essential for successful tissue repair. In addition, cerium modulates key inflammatory pathways, reducing the production of pro-inflammatory cytokines and promoting the transition of macrophages from M1 (inflammatory) to M2 (regenerative), thus supporting the resolution of the dysregulated inflammatory stage characteristic of diabetic wounds. Together, these antioxidant and immunomodulatory actions highlight cerium as a promising therapeutic element for improving and accelerating the healing of chronic diabetic wounds [10,11,12].

Thus, the present study aims to promote the development of bioactive hydrogels, combined with cerium-doped bioactive glasses, in order to overcome the lack of therapeutic response associated with currently available passive hydrogels that do not promote the healing of chronic diabetic wounds. In this sense, the use of cerium-doped bioactive glass allows angiogenic, antioxidant, and anti-inflammatory properties to be included in the hydrogel. This approach is particularly promising for the development of advanced dressings capable of responding to the complex challenges of chronic diabetic wounds.

## 2. Results and Discussion

### 2.1. Bioglass Characterisation

#### 2.1.1. Bioglass Structural Analysis

**Figure 1:**
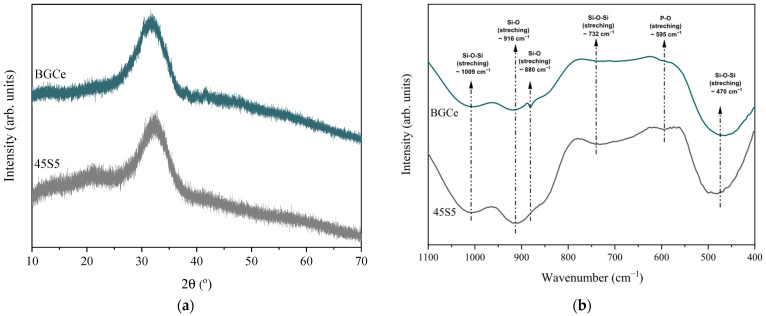
(**a**) XRD patterns of BGCe and Bioglass 45S5; (**b**) FTIR spectra of BGCe and Bioglass 45S5 with the main vibration’s bands.

Structural analysis was performed to assess the effect of adding CeO_2_ to the glass network of Bioglass 45S5. The X-ray diffraction (XRD) spectra of bioglass doped with 5 mol% of CeO_2_ (BGCe) were very similar to those of pure Bioglass 45S5 (45S5), as shown in Figure 1a. This indicates that the introduction of CeO_2_ did not promote the formation of crystalline phases, preserving the characteristic amorphous structure of Bioglass 45S5. The spectrum of Bioglass 45S5 was used as a control for comparison, and the results are in line with previous studies [13,14,15]. The preservation of the amorphous structure of bioactive glass is essential due to its importance in the degradation rate and bioactivity. The typical bioactivity of these materials depends on the glass’s ability to interact with the surrounding physiological environment and tissues and release bioactive ions through surface reactivity and partial dissolution of the glass network. In contrast to crystalline biomaterials (i.e., hydroxyapatite), the amorphous network dissolves more easily in physiological fluids, allowing a continuous and controlled release of therapeutic ions. The ions released from Bioglass 45S5, such as Ca^2+^, Na^+^, Si^4+^, and P^5+^, stimulate angiogenesis, enhance cell proliferation and migration, and promote collagen deposition, thereby accelerating skin regeneration and wound healing. Furthermore, when doped with cerium, the material can release Ce ions capable of interacting with reactive oxygen species (ROS), thus reducing oxidative stress and modulating inflammatory processes [10]. Therefore, maintaining the amorphous structure ensures the therapeutic performance of bioglass, especially in applications related to wound healing where controlled release of therapeutic agents is required [16].

FTIR analysis was also performed to assess the incorporation of CeO_2_ into the glass network. Figure 1b shows the FTIR spectra of BGCe and Bioglass 45S5 (used as a reference). The bands centred at approximately 1009, 916, 880, 732, 595 and 472 cm^−1^ in both spectra are characteristic of Bioglass 45S5. The peak observed at 1009 cm^−1^ corresponds to the asymmetric stretching mode of the Si–O–Si bond; the peaks at 916 cm^−1^ and 880 cm^−1^ are associated with the stretching mode of the Si–O non-bridging oxygen atoms. The band centred at 732 cm^−1^ is related to the Si–O–Si symmetrical stretching mode, while the peak at 472 cm^−1^ refers to the Si–O–Si symmetrical bending mode. The absorption band at 595 cm^−1^ corresponds to the bending mode of the P–O bond [13,17]. The results showed the absence of new vibration bands associated with interactions with cerium or crystalline phases in the BGCe sample compared to the reference (45S5), suggesting that cerium is present in the glass network as a network modifier without interfering with the forming network. Previous studies have shown that the insertion of cerium into silicate glasses up to at least 5 mol% shows no evidence of the formation of new vibration bands [16].

#### 2.1.2. Bioglass Morphological Analysis

The average particle size of BGCe was determined through granulometric analysis. Particle size plays a crucial role in influencing the distribution and dispersion of bioglass within the hydrogel matrix. In addition to greater distribution in bioglass, smaller particles are advantageous for increasing the therapeutic effect of the hydrogel in the wound healing process, as they allow greater interaction with the environment and, consequently, greater release of ions into the surrounding tissues [18].

**Figure 2:**
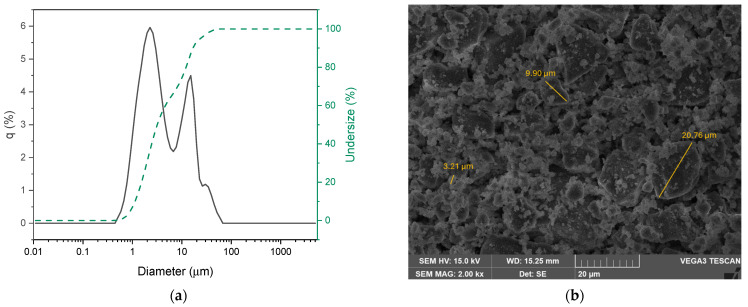
(**a**) Particle size distribution of BGCe by laser diffraction. (**b**) SEM micrograph of BGCe particles (magnification 2 kx).

As can be seen in Figure 2a, the BGCe powder has a wide size distribution, which may be associated with the time conditions in the ball milling method. In addition, these results may be associated with particle agglomeration during the process due to the hygroscopicity of the material. Three distinct size groups can be identified: 10% undersize 1.13 µm, 50% undersize 3.19 µm and 90% undersize 16.82 µm. The mean size was 7.00 µm ± 8.25 µm, and the maximum size was 58.90 µm. Despite the bioglass having been sieved using a 45 μm mesh, this maximum suggests that the sieving process in a vibrating sieve or the storage conditions promoted the particles agglomeration. To obtain a more uniform size distribution and smaller sizes, the milling duration (longer), the diameter of the balls (small balls) and the grinding mode (wet mode) can be adjusted [18]. Although most developed hydrogels in the field of cutaneous regeneration contain bioglass particles with nanometric dimensions, there is also evidence supporting the successful incorporation of Bioglass 45S5 particles with sizes ranging from 2 to 10 µm. Therefore, the developed hydrogel holds therapeutic potential for tissue regeneration applications [19].

SEM image of the BGCe powder and the particle diameter, obtained using ImageJ software (Version 1.54p), are also illustrated in Figure 2b. The image reveals that the particles have irregular shapes and confirms a wide size distribution. The morphology analysis confirmed this heterogeneous distribution and suggested that some of the small particle formed agglomerates, which could influence the results of the laser scattering. Previous studies have verified the influence of ball milling time on iron-containing bioglass. The laser diffraction and SEM results showed that 48 h milling cycles with 5 mm diameter balls promote particle homogeneity, shape the particles from irregular to spherical morphology and reduce the size distribution The study demonstrated that under these conditions it is possible to achieve average diameters of approximately 2 µm [20].

**Figure 3:**
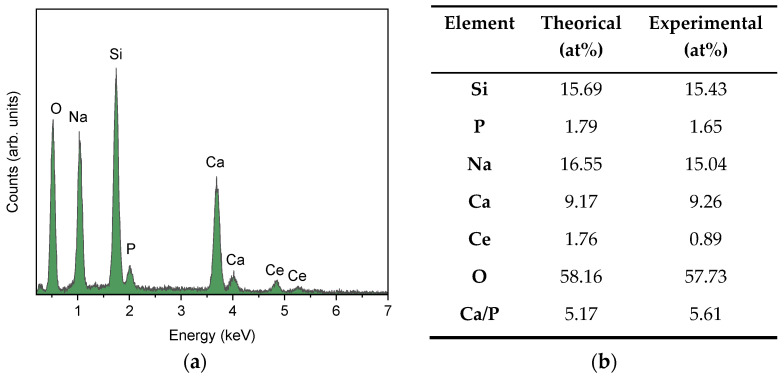
(**a**) EDS spectrum of BGCe; (**b**) Theoretical and experimental atomic percentage of the Na, Si, Ca, P, Ce, O, and Ca/P ratio.

The EDS results for the acquired image area and the theoretical and experimental values for the atomic percentage of each element are shown in Figure 3a,b, respectively. The EDS spectrum reveals the presence of all the main constituents of the bioglass formulation, without contaminations. The detection of cerium confirms that the dopant was successfully incorporated into the glass matrix, but at a lower concentration than expected. This difference may be related to the loss of CeO_2_ in the bioglass synthesis process, which occurs due to the sedimentation of excess cerium at the bottom of the crucible during the melting process. This may be due to the higher atomic weight of the element compared to the other elements present in the formulation and also to the higher density of CeO_2_. The remaining elements have atomic percentages very close to those expected, and the ratio between Ca and P, typical of the Bioglass 45S5 formulation, is close to 5.17 [21].

### 2.2. Hydrogel Characterisation

#### 2.2.1. Hydrogel Structural Analysis

**Figure 4:**
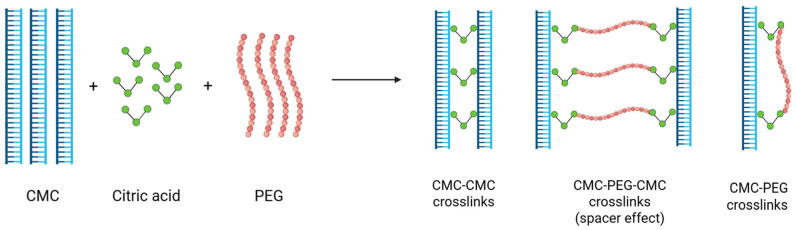
Schematic representation of the crosslinking mechanism in CMC/PEG hydrogels where the citric acid acts as a crosslinking agent between CMC and PEG (Created in BioRender. Marques, I. (2025). https://BioRender.com/kt3xyvd).

In this study, a CMC-PEG hydrogel was developed using citric acid as the crosslinking agent, into which cerium-doped bioglass was incorporated. The combination of CMC and PEG with the citric acid results in hydrogen bonding and ester linkage between the hydroxyl groups (–OH) of PEG and the carboxylate groups (–COO^−^) of CMC (Figure 4). PEG contributes to a more rigid and chemically stable network. Moreover, its spacer effect within the polymer matrix allows a controlled and long-term release of therapeutic agents [22]. Thus, CMC-PEG hydrogel represents a highly functional and versatile system with tailored properties for tissue regeneration and wound healing applications [23,24].

**Figure 5:**
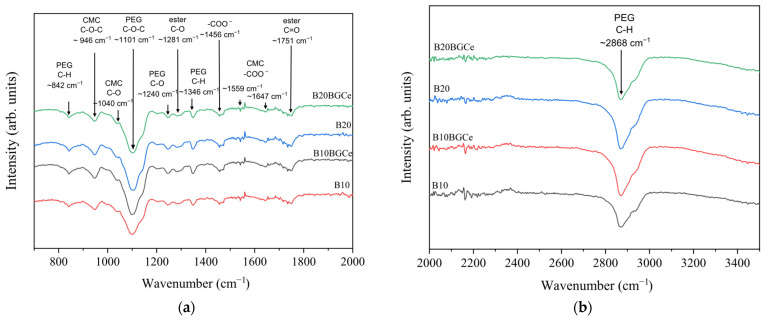
FTIR spectra of B10, B10BGCe, B20 and B20BGCe hydrogels (**a**) in the region of 700–2000 cm^−1^ and (**b**) in the region of 2000–3500 cm^−1^.

Figure 5 shows the FTIR spectra of hydrogels containing 10% and 20% (*w*/*w*) citric acid (BG10 and BG20, respectively), as well as those incorporating 5% (*w*/*w*) BGCe (BG10BGCe and BG20BGCe). The characteristic absorption bands of CMC were observed at 946, 1040, 1559, and 1647 cm^−1^. The vibration modes centred at 1040 cm^−1^ is associated with C–O stretching, and 1559 and 1647 cm^−1^ are related to asymmetric stretching of –COO^−^. The absorption bands also show β1-4 glycoside bonds (C–O–C) between glucose units at 946 cm^−1^. The presence of PEG showed broad bands centred at 1101 cm^−1^ associated with C–O–C stretching mode, and at 2868 cm^−1^ related to symmetric C–H stretching. Moreover, the peaks centred at 1346 and 842 cm^−1^ corresponds to C–H bending modes and at 1240 cm^−1^ is associated with asymmetric stretching mode of C–O. The vibration mode centred at 1751 cm^−1^ indicates C=O ester bonds, and the absorption band at 1281 cm^−1^ is associated with the stretching of C–O ester bonds, as a result of the crosslinking reactions. The peak at 1456 cm^−1^ results from the combining contributions of C–H bending mode from PEG chains and symmetric –COO^−^ stretching of carboxylates [22,23,24,25]. The FTIR spectra showed that the structural integrity of the CMC and PEG in the hydrogel network was preserved. The presence of ester bonds proved the effectiveness of citric acid in the crosslinking reactions, developing a stable structure. Furthermore, the bioglass incorporation did not compromise the bonding in the polymer matrix since new bands were not observed in the B10BGCe and B20BGCe spectra. These findings are attributed to the relatively low concentration of bioglass compared to the polymers, along with the overlap between the polymers’ characteristic bands and the bioglass bands, which prevented its detection [26].

#### 2.2.2. Hydrogel Morphological Analysis

Morphological analysis was performed to evaluate the surface topography and porosity of the hydrogel and the distribution of bioglass in the hydrogel matrix. Porosity plays a key role in enhancing water retention, ion and drug release, and cell interactions, all of which are essential for tissue regeneration. However, larger pores, while promoting healing, may reduce mechanical strength and accelerate degradation [27,28].

**Figure 6:**
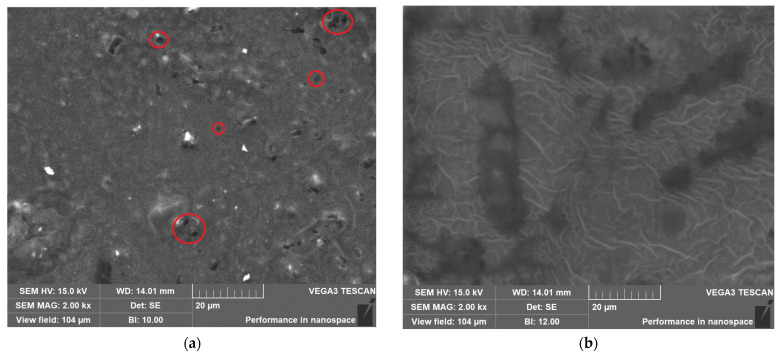
SEM image of (**a**) B10 (red circles = porous area) and (**b**) B20 at magnification of 2 kx.

SEM images of the B10 and B20 hydrogels are represented in Figure 6. The B10 hydrogel exhibits some visible pores distributed across the matrix, which can be identified by the darker points (red circles in Figure 6a). In contrast, the B20 revealed a more compact network with several striations on the surface. These morphological differences can be associated with the higher concentration of citric acid, which rise the crosslinking, leading to a more rigid network [29].

**Figure 7:**
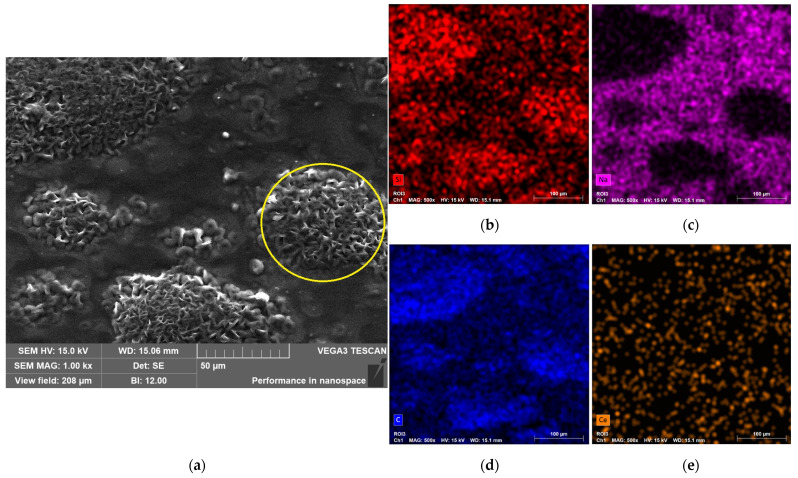
(**a**) SEM image of B10BGCe at magnification of 1 kx (yellow circles = agglomerated particles); (**b**) Elemental mapping of Si; (**c**) Elemental mapping of Na; (**d**) Elemental mapping of C; (**e**) Elemental mapping of Ce.

Figure 7a presents the SEM image of the B10BGCe hydrogel, along with the corresponding elemental mapping images (Figure 7b–e). The SEM micrograph reveals a heterogeneous surface morphology with agglomerated particles (yellow circles) distributed throughout the hydrogel matrix. Elemental mapping indicates that Na is unevenly distributed, showing higher concentrations around the clusters, whereas Si and C appear to be more concentrated within the clusters themselves. Sodium mainly originates from the CMC, with some contribution from BGCe, while silicon is associated with the network former structure of BGCe. Despite the identification of two distinct sodium- and silicon-rich regions, cerium is homogeneously dispersed across the polymer matrix, as shown in Figure 7e. During hydrogel synthesis, the bioglass particles are incorporated into the matrix; however, the distinct distribution of Si and Ce, despite both originating from BGCe, suggests that partial dissolution of the glass occurred, leading to a separation of its components within the hydrogel. The extended magnetic stirring time may have accelerated bioglass dissolution, favouring interactions between the released BGCe ions and the polymer network [30,31].

**Figure 8:**
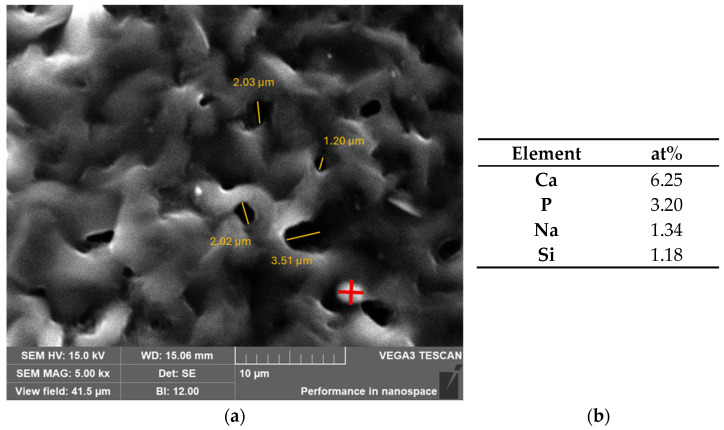
(**a**) SEM image of B10BGCe at magnification of 5 kx (red symbol = particle rich in Ca and P); (**b**) Atomic percentage of Ca, P, Na, Si in the marked area by the red cross.

Figure 8a is the SEM image of the B10BGCe hydrogel at 5 kx magnification, providing a more detailed view of the agglomerates identified in Figure 7a. The micrograph shows a porous microstructure, and some pores were identified in the image. The average pore size was obtained using ImageJ software. The average pore size was 2.03 µm ± 0.83 µm. A study revealed that the presence of pores, with dimensions of 2–3 µm, in a hydrogel promoted cell migration and fibroblast proliferation, thus increasing tissue re-epithelialization [32]. SEM micrographs also revealed the presence of spherical particles in this region. An EDS analysis (Figure 8b) was performed on the particle shown in Figure 8a. The results revealed that part of the bioglass was dissolved in the hydrogel matrix, resulting in particles rich in Ca and P, which confirms the previous discussion.

**Figure 9:**
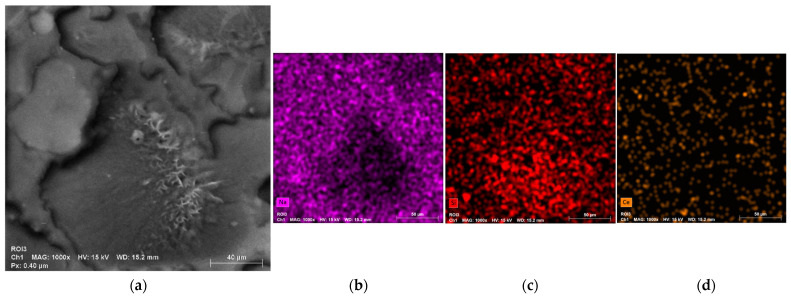
(**a**) SEM images of B20BGCe at magnification of 1 kx; (**b**) Elemental mapping of Na; (**c**) Elemental mapping of Si; (**d**) Elemental mapping of Ce.

The SEM image of B20BGCe, shown in Figure 9a, showed significant differences compared to B10BGCe. In B20BGCe, a relatively denser and more compact surface can be observed, with fewer pores and rougher regions. The reduced number of pores observed may be associated with a higher degree of crosslinking resulting from the increased citric acid concentration. This increase in the degree of crosslinking limits the hydrogel’s ability to absorb and retain water, as further corroborated by the swelling test results presented later [33].

The elemental mapping of Si revealed localised agglomeration in rough areas, while Na exhibited a dispersed distribution around the Si-rich phase, as shown in Figure 9b,c. This distribution is consistent with that observed in the B10BGCe hydrogel. Similarly, Ce also showed a homogeneous dispersion throughout the hydrogel matrix (Figure 9d).

### 2.3. Physical, Biological and Mechanical Properties

#### 2.3.1. Swelling Ratio and Degradation Rate

Swelling tests were performed to evaluate the water absorption and retention capacity of the hydrogels and the influence of citric acid concentration and bioglass incorporation on their swelling ratios. The highly crosslinking level creates a resistance network restricting water diffusion into the matrix. In this way, increasing the concentration of crosslinking agents promotes improved structural stability but compromises water retention [34].

**Figure 10:**
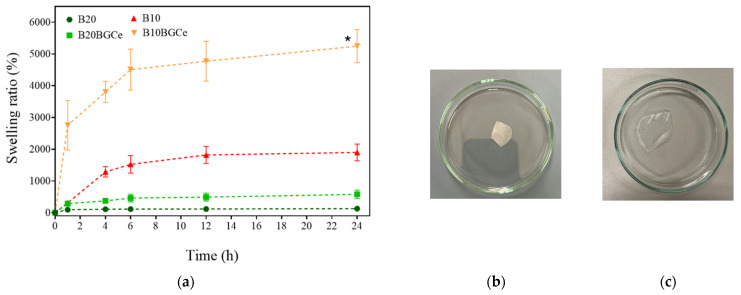
(**a**) Swelling ratio of B10, B10BGCe, B20, and B20BGCe hydrogels measured after 1, 4, 6, 12, and 24 h. Data are presented as mean ± standard deviation (n = 3); (**b**) B20 hydrogel before and (**c**) after 1 h of the swelling test. * Result compromised.

Figure 10a illustrates the swelling behaviour of all hydrogels, whereas Figure 10b,c show representative images of the B20 hydrogel before immersion in water and after 1 h of swelling, respectively. B10BGCe exhibited a significantly greater swelling ratio compared to the other samples. This results’ discrepancy was attributed to the transition of the hydrogel into a gelatinous stage, which compromised the effective removal of the excess water. In this way, the measures of the final weight (wf) have been overestimated, compromising the results. The reduced crosslinking degree, associated with the heterogeneity of the matrix observed in the SEM images (Figure 8a), may explain the poor structural stability and the gelatinous behaviour of B10BGCe. Therefore, this sample was not considered in the following discussion. The B10 sample achieved a maximum swelling ratio of around 1900% after 12 h. Initially, after 4 h, this hydrogel showed a remarkable increase in the swelling capacity, followed by a gradual stabilisation, which achieved equilibrium after 6 h. Conversely, B20 did not show significant differences in the swelling rate over time, remaining nearly constant. This evidence suggests that a higher concentration of crosslinking agent promotes the formation of covalent bonds between the functional groups of the polymer chains decreasing the swelling ratio [23]. Moreover, the addition of the bioactive glass to this hydrogel showed an increased in the swelling ratio. In B20BGCe, the swelling ratio smoothly increased from a minimum of 285% to a maximum of 575%, reaching equilibrium after 6 h. These results suggest that the bioglass may have competed with the citric acid bonds to the polymers, decreasing the crosslinking degree and leading to a greater swelling. This behaviour contrasts with the findings reported by Zheng et al. [35], where the incorporation of bioactive glass led to a decrease in swelling capacity.

The degradation test was performed to evaluate the structural stability of the hydrogels and analyse the impact of citric acid concentration and BGCe incorporation on the degradation rate. The therapeutic efficacy of hydrogels in biomedical applications is directly related to their degradation rate, which must be controlled. Ideally, the degradation rate of the hydrogel should be compatible with tissue regeneration, without compromising wound healing and without inducing an inflammatory response. Slower degradation rates can delay healing, while excessive degradation rates lead to uncontrolled drug release, increasing the risk of local toxicity and potential inflammatory response [36].

**Figure 11:**
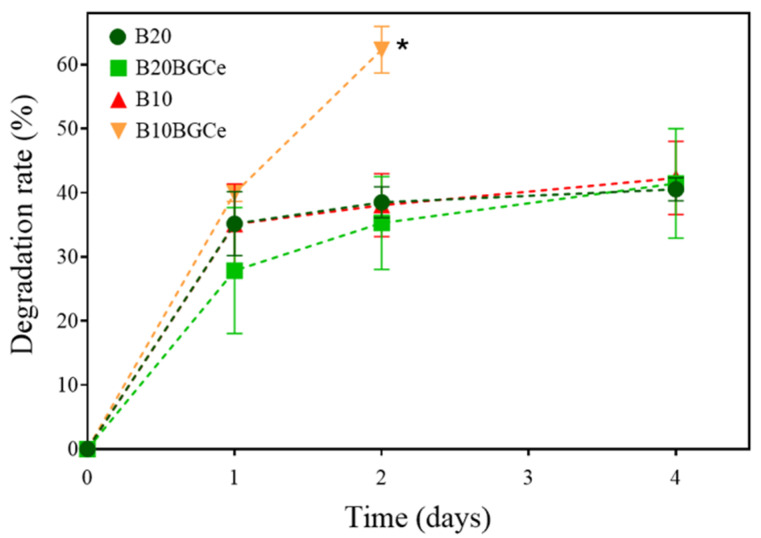
Degradation rate (%) of B10, B10BGCe, B20, and B20BGCe hydrogels measured after 1, 2, and 4 days. Data are presented as mean ± standard deviation (n = 3). * Completely dissolved.

In Figure 11 it is possible to analyse the degradation test results. Against what was expected, B10 and B20 did not show a significant difference in the degradation rate [23]. Both samples exhibited an increased initial degradation, followed by a gradual stabilisation, reaching a maximum value of around 40%. B20BGCe showed a slightly inferior degradation rate after 1 day. This behaviour suggested that the incorporation of bioglass within the hydrogel network enhances the resistance and stability, improving the material’s mechanical properties in an early phase [19]. Nevertheless, after 2 days, the degradation behaviour was comparable to that of the other samples, reaching the same maximum value. The B10BGCe exhibited a degradation rate significantly higher. After 2 days, the hydrogel network lost its structural integrity and dissolved, so the degradation test did not proceed. This behaviour is beneficial for dressing applications that require an intensive drug release in an early stage. The increased degradation rate enables the effective and targeted delivery of therapeutic agents to the wound site, which can accelerate and improve the healing process during the initial phases of treatment. Nevertheless, the lack of structural stability in the B10BGCe may limit its use for long-term applications, particularly in wounds that require sustained release of bioactive ions [37].

#### 2.3.2. Biocompatibility

**Figure 12:**
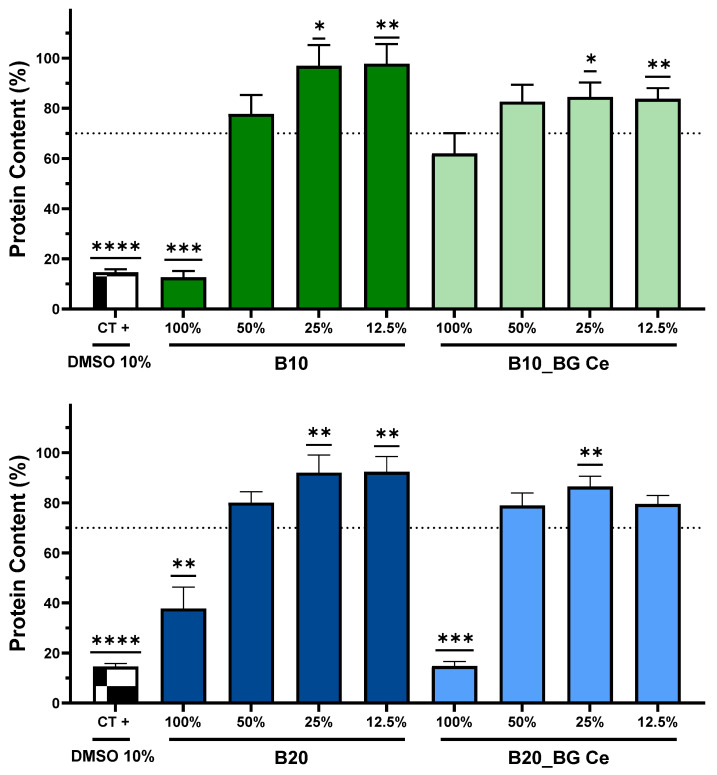
Protein content of Vero cells after 48 h of incubation with different extract concentrations (100%, 50%, 25%, and 12.5%) of B10, B10BGCe, B20, and B20BGCe hydrogels. Protein content was measured through SRB assay with results being normalised to control (non-treated cells) and presented as mean ± standard error of mean of, at least, three independent experiments in duplicate. The results were statistically compared to the hypothetical value of 70% (threshold for cytotoxicity as ISO 10993:2009-5) using the non-parametric one-sample Wilcoxon test; (* *p* < 0.05; ** *p* < 0.01; *** *p* < 0.001; **** *p* < 0.0001).

The biocompatibility of the hydrogels, either with (B10BGCe and B20BGCe) or without (B10 and B20) the incorporation of bioglass dopped with cerium, was assessed based on protein content using the SRB assay. The effects on Vero cells protein content, after 48 h of incubation with hydrogels extracts at different concentrations (100%, 50%, 25%, and 12.5%), are presented in Figure 12. According to ISO 10993:2009-5 “Biological evaluation of medical devices—Part 5: Tests for in vitro cytotoxicity” guidelines, a significant reduction in protein content exceeding 70% is considered indicative of cytotoxicity.

The exposure of Vero cells to a 100% (undiluted) B10 hydrogel extract concentration induced a reduction in protein content to 12.65 ± 2.43%, significantly below the ISO cytotoxicity threshold of 70% (*p* = 0,001). However, no cytotoxic effects were observed in Vero cells when exposed to 50%, 25%, and 12.5% B10 hydrogel extract dilutions, as protein content values remain above 70%. When bioglass dopped with cerium was incorporated into B10 hydrogels (B10BGCe), none of the tested extract concentrations induced cytotoxicity, as Vero cells’ protein content remained above the 70% threshold in all tested conditions. Regarding the B20 hydrogels, the 100% extract (undiluted) induced a decrease in protein content of Vero cells to 18.54 ± 8.53%, which is significantly (*p* = 0.0098) below the ISO cytotoxicity threshold of 70%. Despite the cytotoxicity at the undiluted extract concentration, no cytotoxic effect was observed at 50%, 25%, and 12.5% concentration. When bioglass dopped with cerium was incorporated into B20 hydrogels (B20BGCe), the undiluted extract (100%) was shown to be cytotoxic due to the reduction in Vero cells’ protein content to 10.28 ± 1.81%, which is significantly lower than 70% (*p* = 0.001). All other tested B20BGCe extract concentrations were shown to be non-cytotoxic. The sensitivity and reliability of the assay were confirmed using positive control (10% DMSO), which induced a significant reduction in protein content of Vero cells to below the 70% threshold (*p* < 0.0001).

Collectively, these results demonstrate that both B10 and B20 hydrogels exhibit extract concentration-dependent cytotoxicity, with significant reductions in Vero cell protein content observed only at the undiluted (100%) extract concentration. At lower extract concentrations (≤50%), no cytotoxic effects were observed, with protein levels being above 70%. The incorporation of cerium-doped bioglass into the B10 hydrogel formulation reduced its cytotoxicity, including at the undiluted concentration. These findings are consistent with those reported by Rigamonti et al. [38], which indicated that bioactive glasses doped with low concentrations of cerium are non-cytotoxic. This effect can be explained by the redox properties of cerium, which can reversibly switch between Ce^3+^ and Ce^4+^ oxidation states, enabling the scavenging of reactive oxygen species (ROS). This antioxidant activity mitigates oxidative stress generated by reactive components within the hydrogel matrix, thereby protecting cells and contributing to its reduced cytotoxicity [38,39].

The significant cytotoxicity observed with undiluted extracts of B10, B20, and B20BGCe may be attributed to the release of acidic by-products from the crosslinking agent or other soluble degradation products, which become more concentrated in the undiluted extracts. These components can lead to local acidification, impairing cellular metabolism, integrity, and protein synthesis, considering the optimal pH range for Vero cell growth is between 7.0 and 7.4 [40]. However, the absence of cytotoxicity at extract dilutions of 50% or lower suggests that these components are effectively diluted below harmful levels, thereby providing a favourable microenvironment for cell maintenance and proliferation, and supporting the overall biocompatibility of the materials. Overall, the incorporation of cerium-doped bioglass appears to enhance the biocompatibility of B10 hydrogels without affecting material stability, potentially due to the antioxidant and redox-modulating properties of cerium ions.

#### 2.3.3. Bending Strength

The hydrogel’s mechanical resistance was evaluated through cyclic bending tests performed on porcine skin to mimic the successive skin movements, particularly at flexion areas and articulations, a resistant material is essential. This assay was exclusively performed on the B10 and B10BGCe samples, as they were the only formulations that exhibited no cytotoxic effects.

**Figure 13:**
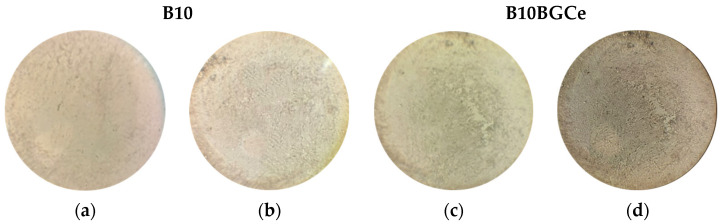
Optical microscope images of the B10 hydrogel network before (**a**) and after (**b**) the bending tests and B10BGCe hydrogel network before (**c**) and after (**d**) bending tests.

The results are presented in Figure 13. After the consecutive deformations over the 250 cycles, none of the samples revealed any microfissure formations. In B10BGCe, a non-homogeneous region in the network was observed before the bending test. However, this defect did not compromise the network integrity during the cycles since new microfissures were not formed in the affected area. In this way, the results reveal that the mechanical stability of the hydrogels after consecutive deformation was preserved without compromising their integrity. Moreover, throughout the test, great hydrogel adhesion to the porcine skin was observed without detachment or sliding, revealing the potential of these hydrogels as flexible wound dressings.

## 3. Conclusions

The main objective of this project was to develop a multifunctional hydrogel doped with cerium oxide for tissue regeneration. The XRD patterns and FTIR results of BGCe did not reveal the formation of crystalline phases, proving that the addition of Ce did not compromise the structure of Bioglass 45S5. The SEM and granulometric data indicated that there were some agglomerates and that more than 90% of the particles were less than 18 microns in diameter.

Considering the hydrogels, the FTIR spectra showed the characteristic vibration bands of polymers and ester bonds resulting from the cross-linking processes, proving that the hydrogel structure was not affected by the addition of BGCe. The SEM images of the hydrogels reveal morphological changes in the structure and a possible dissolution of BGCe in the matrix. The B10BGCe hydrogel presented a porous network with elemental homogeneity, while the B20BGCe hydrogel revealed a denser and more compact structure. In both samples, it was possible to identify the presence of bioglass and cerium dispersed very uniformly.

Swelling tests reveal that the sample with the highest acid concentration promotes less water retention in the hydrogel matrix. In degradation tests, the samples showed a similar degradation rate, except for sample B10BGCe, which degraded more rapidly after 24 h of immersion. However, cytotoxicity tests proved that sample B10BGCe was completely non-toxic at all concentrations of the extract. The analysis of the mechanical properties reveals that the developed hydrogels present high deformation resistance, which is crucial for wound dressings. These results allowed the selection of the B10BGCe formulation as the most promising option for hydrogel applications.

Further studies evaluating the quantification of degradation by-products, prolonged exposure of cells to these products, and a comprehensive assessment of antioxidant effects will be crucial to confirm long-term cytocompatibility and clarify the mechanisms underlying cerium-mediated antioxidant effects. Furthermore, future work should explore the wound healing potential and other functional properties using more relevant cell types, such as keratinocytes and fibroblasts, as well as advanced 3D skin cell cultures.

## 4. Materials and Methods

### 4.1. Materials

The starting materials used for the synthesis of the cerium-doped bioactive glass were silica (SiO_2_), calcium carbonate (CaCO_3_), sodium carbonate (Na_2_CO_3_), phosphorus pentoxide (P_2_O_5_), and cerium (IV) oxide (CeO_2_), all supplied by Merck KGaA (Darmstadt, Germany). For the hydrogel synthesis, polyethylene glycol 2000 (PEG 2000), carboxymethyl cellulose sodium salt (CMC, average molecular weight: 250,000 Da), and citric acid (CA) were used, also obtained from Merck KGaA (Darmstadt, Germany).

### 4.2. Synthesis of Bioglass 45S5 Doped with CeO_2_

In this work, a Bioglass 45S5 with CeO_2_ (5 mol%) was developed by the melt-quenching technique. The proportion of Bioglass 45S5 components was maintained while the CeO_2_ were introduced (BGCe) (Table 1).

**Figure 14:**
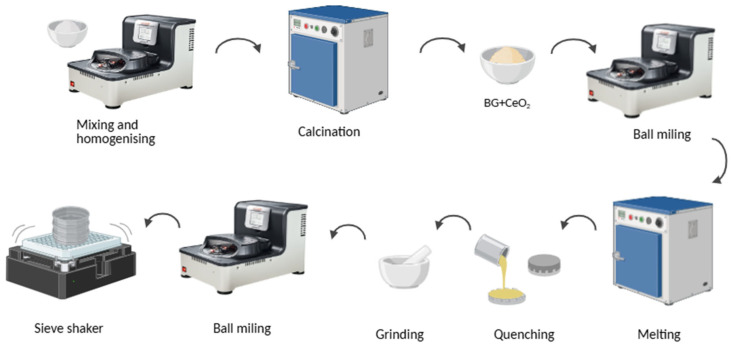
Illustrative scheme of bioglass synthesis steps (created in BioRender. Pacheco, S. (2025) https://BioRender.com/2sgv3fp).

As illustrate in Figure 14, the process started with the mixing and homogenising of the starting reagents, SiO_2_, CaCO_3_, Na_2_CO_3_ and P_2_O_5_ through a planetary ball milling (Fritsch Pulverisette 5, FRITSCH GmbH, Idar-Oberstein, Germany) for 1 h at 300 rpm. In this process, two agata vessels of 250 mL and 40 agata balls with a diameter of 10 mm were used. Subsequently, the mixture was calcined in an alumina crucible at 800 °C for 8 h, using a heating rate of 10 °C/min, to remove the carbon dioxide (CO_2_) from the initial reagents.

Once the calcination process was completed, the CeO_2_ were added to the sample and the mixture was homogenised using the same planetary ball milling system for 1 h at 300 rpm. Afterwards, the sample was melted in a platinum crucible at 1350 °C for 1 h. To promote a more homogeneous element’s distribution in the network the bioactive glass was re-melted under the same conditions for 2 cycles. The melted glass was poured into a metallic mould, at room temperature, to induce the thermal shock and pressure with a metal piece to weaken the glass. The resulting bulk glass was manually ground in an agata mortar and milled in the high-speed planetary ball milling (Fritsch Pulverisette 7) for 8 h at 500 rpm (30 min running with 45 min break to avoid overheating). Finally, the BGCe powder was sieved using a Scansi AS 200 Basic (Retsch, Haan, Germany) sieve shaker with a 45 µm mesh. This process was performed to select the smallest particle dimensions for subsequent incorporation into the hydrogel.

### 4.3. Synthesis of Bioglass-Containing Hydrogels

The hydrogels were developed using carboxymethyl cellulose sodium salt (CMC) 250,000 (average molecular weight: 250,000 Da), polyethylene glycol (PEG 2000), and citric acid (CA). CA was added at 0, 10 and 20% by weight relative to the total weight of the other components (CMC and PEG). The several formulations are represented in Table 2.

Cellulose was chosen as the base material for the hydrogel formulation due to its natural abundance, low cost, biocompatibility, biodegradability, and environmental friendliness. Specifically, carboxymethyl cellulose, a water-soluble, polyanionic derivative of cellulose rich in carboxymethyl groups, was selected because these functional groups provide excellent water absorption capacity and confer desirable viscoelastic properties to the hydrogel. The lower molecular weight CMC (250,000 Da) produces more flexible but mechanically weaker gels. To enhance the matrix further, polyethylene glycol (PEG) was incorporated as a synthetic amphiphilic polymer. PEG’s biocompatibility and non-immunogenic nature, combined with its ability to create spacing between polymer chains, improve the swelling behaviour of the hydrogel and facilitate effective drug incorporation [23]. The citric acid was used as a crosslinking agent in different concentrations.

**Figure 15:**
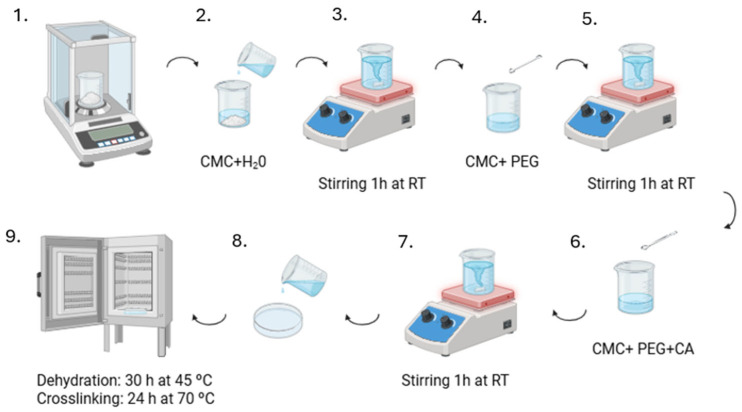
Schematic hydrogel synthesis procedure without CeO_2_-doped bioglass (created in BioRender. Pacheco, S. (2025) https://BioRender.com/f66dsc5).

Initially, hydrogels were synthesised without the bioglass (as control) to compare with the hydrogels containing bioglass (Figure 15). CMC-PEG hydrogels were prepared based on the previously reported method [23].

CMC solutions were prepared by mixing CMC with 200 mL of deionised water and stirring at room temperature (RT) using a magnetic stirrer until complete dissolution. After dissolution, PEG was added, and the solutions were kept under magnetic stirring at RT for 1 h. Finally, the crosslinking agent, citric acid, was added at concentrations of 10% *w*/*w* and 20% *w*/*w* under magnetic stirring at RT for 1 h. Afterwards, 18 mL of the solutions were poured into Petri dishes and dried at 45 °C for 30 h. Then, the samples were kept at 70 °C for 24 h for the crosslinking reaction.

The hydrogels containing CeO_2_-doped bioglass were prepared using the same method described above. The BGCe was added after mixing the CMC and PEG (step 5 shown in Figure 15). The BGCe powder (∅ < 45 µm) was added (5% by mass relative to the total mass of CMC and PEG) to the solution under magnetic stirring at room temperature for 1 h. After BGCe incorporation, CA was added (step 6) and the following steps were performed according to the scheme in Figure 15. The samples were designated B10BGCe and B20BGCe.

### 4.4. Materials Characterisation

#### 4.4.1. Characterisation of the Bioglass

The structural characterisation of the BGCe was analyse by X-ray diffraction (XRD) and Fourier-transform infrared spectroscopy (FTIR).

X-ray diffraction was performed to verify if the addition of the CeO_2_ promotes the formation of crystalline phases in the Bioglass 45S5 structure. X-ray diffraction of the BGCe powder was acquire using Aeris Panalytical diffractometer (Malvern Panalytical B.V., Almelo, The Netherlands) and CuKα radiation (λ = 1.5406 Å, 40 kV, 15 mA) for 1 h. Bioglass 45S5 was also analysed as control.

FTIR was used to characterise the chemical structure of the bioglass and to detect possible changes in the glass network induced by the modified formulation, which included the addition of CeO_2_. FTIR spectra were recorded using a PerkinElmer Spectrum Two (PerkinElmer^®^, Shelton, CT, USA) in the attenuated total reflection mode (ATR). The samples were scanned in the range of 4000–400 cm^−1^ with a resolution of 4 cm^−1^. All the measurement was obtained for BGCe and Bioglass 45S5 to comparation.

The size and morphology of the BGCe particles were evaluated by laser diffraction and scanning electron microscopy (SEM) techniques. In granulometric analysis by laser diffraction was performed using a HORIBA Scientific LA-960 laser diffraction analyser (Horiba Ltd., Irvine, CA, USA), in the wet mode, dispersing the powder in ethanol (1 mg/mL) by ultrasound bath before the measurement. This pattern is analyse using Fraunhofer mathematical model to determine the particle size distribution [41]. SEM was performed to analyse the geometry of the BGCe particles and assess the possible particles agglomeration. The micrographs were obtained using a microscope from TESCAN model Vega 3 (TESCAN ORSAY HOLDING, A.S., Brno-Kohoutovice, Czech Republic). Moreover, the energy dispersive spectroscopy (EDS) was used to evaluate the element’s distribution, verify the expected concentrations and detect potential contamination. The analysis was performed using a Bruker EDS system coupled to the scanning electron microscope.

#### 4.4.2. Characterisation of the Hydrogels

##### Structural and Morphological Characterisation

FTIR analysis was performed to investigate the intermolecular interactions, evaluate the efficiency of crosslinking in the CMC/PEG-based hydrogels, and determine whether the incorporation of bioglass affected the structural bonds of the base hydrogel. Morphological characterisation was performed to evaluate the distribution and integration of the bioglass within the matrix, as well as the topography of the hydrogel network. Both the FTIR and SEM analyses were conducted using the same equipment as those used for the characterisation of the bioglass.

##### Swelling and Degradation Studies

The swelling studied was performed to determine the fluid absorption capacity of the developed hydrogels, which indicated his ability to uptake and retain water or biological fluids at the wound site. The swelling behaviour is an important feature as it enables the hydrogel to absorb large amounts of wound exudate while maintaining a moist environment, which promote cell migration and the wound healing process. Furthermore, swelling enhances the hydrogel’s adhesion to the wound surface. Additionally, the swelling capacity contributes to the efficient retention and controlled release of therapeutic agents [34].

The test was only performed on the hydrogels that completed the crosslinking reactions, with 3 replicates assessed per sample. The dry hydrogels samples were weighted ($w_{0}$) and immersed in deionized water for 1, 4, 6, 12 and 24 h at RT. After each period of time, the swollen hydrogels were removed from the water, carefully dried with filter paper to remove the excess water on the surface, and weighted ($w_{s}$). The swelling ratio was calculated using Equation (1).

(1) $$\%\;swelling\;ratio=\frac{w_{s}-w_{0}}{w_{0}}\times 100$$

The degradation of the hydrogel network has a direct influence on controlled drug release. Therefore, it is crucial to ensure that the degradation rate is synchronised with the pretended biomedical application, as it directly impacts the therapeutic efficacy and the material’s performance. The degradation behaviour was evaluated by determining the weighted loss in deionized water. The dry hydrogels were weighted ($w_{0}$) and immersed in deionized water at 37 °C (body temperature). The samples were taken out from the water after 1, 2, and 4 days of incubation and dried at 40 °C for 24 h. Then, the final weight was measured ($w_{f}$) for each time point. The degradation rate was calculated based on Equation (2). The tests were replicate three times.

(2) $$\%\;degradation\;rate=\frac{w_{0}-w_{f}}{w_{0}}\times 100$$

##### Biocompatibility

To ensure the biocompatibility of the material, cytotoxicity of the hydrogels was evaluated using the Sulforhodamine B (SRB) assay. The assays were performed according to the “ISO 10993-5:2009; Biological evaluation of medical devices—Part 5: Tests for in vitro cytotoxicity” (REF), using the extract method for samples preparation.

For sample preparation, the hydrated hydrogels were first sterilised under UV light for 20 min in a laminar flow cabinet. Then, extracts were prepared at a concentration of 200 mg/mL in Modified Eagle Medium (MEM, Sigma-Aldrich, MO, USA) and incubated for 24 h at 37 °C with 5% of CO_2_ in a humified atmosphere.

Vero cells (CCL-81™, ATCC, Manassas, VA, USA) were seeded in 96-well plates (10,000 cells/well) and left to adhere overnight in the incubator at 37 °C with 5% CO_2_ and 95% air. Then, cells were exposed for 48 h to hydrogel extracts at 100% (undiluted), 50%, 25% and 12.5% dilutions, with 10% dimethyl sulfoxide (DMSO) being used as a positive control and untreated cells as a negative control.

After 48 h, the SRB assay was performed to evaluate cell protein content, as previously described [42]. Briefly, cells were fixed with 1% (*v*/*v*) acetic acid (Sigma 33209, MO, USA) in methanol (Sigma 32213, MO, USA), stained with SRB (Sigma S9012, MO, USA), washed with 1% (*v*/*v*) acetic acid in water to remove excess dye, and solubilized in 10 mM Tris base solution. Absorbance was measured at 540 nm and 690 nm. At least, three independent experiments, in duplicate, were performed for each sample to ensure reproducibility.

Statistical analysis was performed using GraphPad Prism version 10.2.3 for Windows (GraphPad Software, Boston, MA, USA). The non-parametric One sample Wilcoxon test was applied for comparison between each experimental condition and the threshold value of 70%, in accordance with ISO 10993-5:2009 (“Biological evaluation of medical devices—Part 5: Tests for in vitro cytotoxicity”). In accordance, samples were considered cytotoxic when a significant reduction (*p* < 0.05) in protein content below 70% was verified.

##### Mechanical Tests

**Figure 16:**
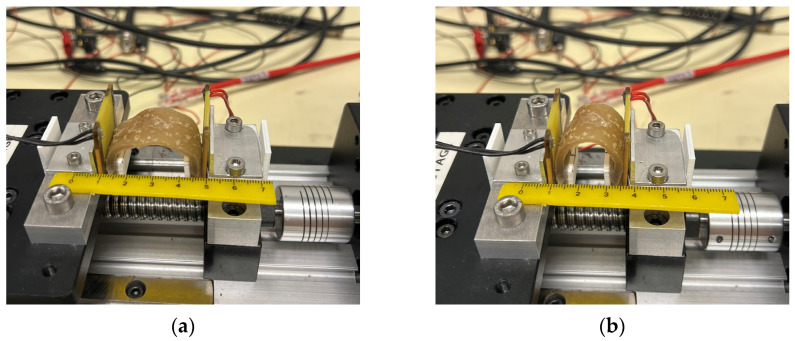
Experimental setup of the cyclic bending test performed on hydrogels applied to porcine skin. (**a**) initial position; (**b**) maximum curvature.

The mechanical properties of the hydrogel network were determined through cyclic bending tests performed on porcine skin, mimicking the dynamic biomechanical conditions of the real cutaneous environment. This assay was conducted using a bending equipment developed at the group’s laboratory (Figure 16). The hydrogel samples were cut into small dimensions and hydrated with deionised water for 1 h at RT. This process ensured adhesion to the skin and simulated the real application conditions since the hydrogel would be applied in its hydrated form. After hydration, the samples were observed in the optical microscope Olympus BH2-UMA with a magnification of 50×. Subsequently, the hydrated hydrogels were applied onto the porcine skin, and bending tests proceeded for 250 cycles at 5 mm/s with a displacement of 14 mm. Finally, the hydrogels were reanalysed on the microscope to assess the formation of possible fissures in the network.

## Figures and Tables

**Table 1:** Composition of Bioglass 45S5 and Bioglass 45S5 doped with CeO_2_ in mol%.

Samples	SiO_2_	P_2_O_5_	CaO	Na_2_O	CeO_2_
Bioglass 45S5 (control)	46.1	2.6	26.9	24.4	-
BGCe	43.8	2.5	25.6	23.1	5

**Table 2:** Formulations of the produced hydrogels.

Samples	CMC (g)	PEG (g)	CA (% *w*/*w*)
B0	3.6	0.4	0
B10			10
B20			20

## Data Availability

The original contributions presented in this study are included in the article. Further inquiries can be directed to the corresponding author.

## Abbreviations

The following abbreviations are used in this manuscript:

ATR	Attenuated total reflection
BG	Bioactive glasses
C	Carbon
CA	Citric acid
Ca	Calcium
CaCO_3_	Calcium carbonate
CaO	Calcium oxide
Ce	Cerium
CeO_2_	Cerium oxide
CMC	Carboxymethyl cellulose
CO_2_	Carbon dioxide
Da	Dalton
DMEM	Dulbecco’s modified eagle medium
DMSO	Dimethyl sulfoxide
EDS	Energy dispersive spectroscopy
FTIR	Fourier-transform infrared spectroscopy
h	Hours
H_2_O	Water
IDF	International Diabetes Federation
min	Minutes
Na	Sodium
Na_2_O	Sodium oxide
P	Phosphorus
P_2_O_5_	Phosphorous pentoxide
PEG	Polyethylene glycol
ROS	Reactive oxygen species
rpm	Rotation per minute
RT	Room temperature
SEM	Scanning electron microscopy
Si	Silicon
SiO_2_	Silica
SiO_4_	Silicate tetrahedron
SRB	Sulforhodamine B
TCA	Trichloroacetic acid
USD	United States dollar
UV	Ultraviolet radiation
XRD	X-ray diffraction
